# Using the *Plasmodium* mitochondrial genome for classifying mixed-species infections and inferring the geographical origin of *P*. *falciparum* parasites imported to the U.S.

**DOI:** 10.1371/journal.pone.0215754

**Published:** 2019-04-30

**Authors:** Sarah E. Schmedes, Dhruviben Patel, Julia Kelley, Venkatachalam Udhayakumar, Eldin Talundzic

**Affiliations:** 1 Malaria Branch, Division of Parasitic Diseases and Malaria, Center for Global Health, Centers for Disease Control and Prevention, Atlanta, Georgia, United States America; 2 Association of Public Health Laboratories, Silver Spring, Maryland, United States America; 3 Williams Consulting LLC, Baltimore, Maryland, United States America; 4 Atlanta Research and Education Foundation, Atlanta, Georgia, United States America; Université Pierre et Marie Curie, FRANCE

## Abstract

The ability to identify mixed-species infections and track the origin of *Plasmodium* parasites can further enhance the development of treatment and prevention recommendations as well as outbreak investigations. Here, we explore the utility of using the full *Plasmodium* mitochondrial genome to classify *Plasmodium* species, detect mixed infections, and infer the geographical origin of imported *P*. *falciparum* parasites to the United States (U.S.). Using the recently developed standardized, high-throughput Malaria Resistance Surveillance (MaRS) protocol, the full *Plasmodium* mitochondrial genomes of 265 malaria cases imported to the U.S. from 2014–2017 were sequenced and analyzed. *P*. *falciparum* infections were found in 94.7% (251/265) of samples. Five percent (14/265) of samples were identified as mixed- *Plasmodium* species or non-*P*. *falciparum*, including *P*. *vivax*, *P*. *malariae*, *P*. *ovale curtisi*, and *P*. *ovale wallikeri*. *P*. *falciparum* mitochondrial haplotypes analysis revealed greater than eighteen percent of samples to have at least two *P*. *falciparum* mitochondrial genome haplotypes, indicating either heteroplasmy or multi-clonal infections. Maximum-likelihood phylogenies of 912 *P*. *falciparum* mitochondrial genomes with known country origin were used to infer the geographical origin of thirteen samples from persons with unknown travel histories as: Africa (country unspecified) (n = 10), Ghana (n = 1), Southeast Asia (n = 1), and the Philippines (n = 1). We demonstrate the utility and current limitations of using the *Plasmodium* mitochondrial genome to classify samples with mixed-infections and infer the geographical origin of imported *P*. *falciparum* malaria cases to the U.S. with unknown travel history.

## Introduction

Malaria is a significant public health problem, with over 3.3 billion individuals at risk of infection worldwide [1]. In 2016, there were 216 million reported cases and 445,000 deaths in 91 countries [2]. Malaria infections in humans are caused by the parasites *Plasmodium falciparum*, *P*. *vivax*, *P*. *ovale*, *P*. *malariae*, and *P*. *knowlesi* [1]. One of the greatest challenges to malaria control and elimination is the rapid spread of drug-resistant infections around the world [2]. In particular, significant effort is now focused on preventing *P*. *falciparum* parasites resistant to first-line artemisinin-combination therapies (ACTs) from further spreading from the Greater Mekong Region [3]. Molecular surveillance of *P*. *falciparum* drug resistance markers greatly facilitates tracking of drug-resistant parasites.

In 2014, there were 1,724 domestically-imported, travel-related cases of malaria in the United States (U.S.) reported to CDC, of which 137 samples were sent to the Centers for Drug Control and Prevention (CDC) for drug-resistance testing [1]. Of the samples tested at the CDC, greater than 95% of samples carried at least one drug-resistance marker [1]. Current methods for *Plasmodium* species and drug-resistance detection include real-time polymerase chain reaction (PCR) and Sanger sequencing, which are time consuming and costly. Recently, we developed a standardized next-generation sequencing and bioinformatics pipeline for Malaria Resistance Surveillance (MaRS), which deep sequences and analyzes, in a single assay, all validated *P*. *falciparum* drug-resistance associated single-nucleotide polymorphisms (and novel variants) in the major drug resistance genes (*crt*, *mdr1*, *k13*, *dhfr*, *dhps*, and *cytochrome b*), as well as the *Plasmodium* mitochondrial genome [4].

While the primary purpose of MaRS is to identify drug resistance, the sequence data generated could provide additional information about imported parasites. For example, sequence data could be used to identify mixed infections and could also help determine the geographic origin of drug-resistant parasites, which would be useful to refine chemoprophylaxis and treatment recommendations as well as for case and outbreak investigations. The *Plasmodium* mitochondrial genome can be used for such purposes, as an additional marker for surveillance. The 6-kb mitochondrial genome is inherited solely from the female gametocyte and does not undergo recombination, making the mitochondrial genome a desirable candidate marker for pathogen surveillance [5–7]. The full mitochondrial genome has been sequenced and used to determine the origin of *P*. *falciparum* from sub-Saharan Africa with multiple introductions to South America and Southeast Asia, depicting distinct population structure [6,8,9]. Furthermore, Preston *et al*. [7] identified 23 SNPs from extrachromosomal DNA, including 5 SNPs from the mitochondrial genome, that could be used to predict geographical origin of *P*. *falciparum* isolates from five regions including South America, West Africa, East Africa, Southeast Asia, and Oceania.

Here, we attempted to explore the potential use of the full 6-kb *Plasmodium* mitochondrial genome using a targeted-amplicon deep sequencing approach for malaria surveillance purposes. MaRS was used to sequence full *Plasmodium* species mitochondrial genomes from U.S. domestically-imported malaria samples, from 2014–2017, as part of an ongoing effort for anti-malarial drug resistance surveillance (see [4] for the corresponding drug-resistance data). The full mitochondrial genome was utilized to identify *Plasmodium* species, detect multiplicity of infection of multiple clonal populations or species in mixed infections, and infer geographical origin of the parasite when travel history is unknown.

## Materials and methods

### Samples and ethics statement

Whole-blood samples (n = 265) were obtained from U.S. domestic, imported malaria cases during 2014–2017 from the CDC domestic malaria surveillance network and tested in accordance with protocol 2017–309 approved by the Office of the Associate Director of Science, Center for Global Health, Centers for Disease Control and Prevention as a surveillance activity.

### Sample prep, library prep, and targeted deep-amplicon sequencing

Detailed methods for DNA extraction, targeted gene enrichment, library preparation, and sequencing were previously reported by Talundzic et al. [4] (also detailed at [10]). Briefly, total DNA was extracted from whole-blood samples using the QIAamp DNA blood kit (Qiagen, USA). The *crt*, *mdr1*, *k13*, *dhfr*, *dhps*, and *cytochrome b* genes and full mitochondrial genome were amplified, normalized, and pooled into one amplicon pool per patient. Libraries were constructed using the Nextera XT kit (Illumina, USA) and indexed at the patient level. Each indexed library was pooled and sequenced on the MiSeq (Illumina) using a 2 x 250 bp read length.

### Sequence alignment, variant and haplotype identification

Fastq files were imported into Geneious v11.0.5 (Biomatters) for analysis. Paired-end sequences were paired, and BBduk Trimmer v37.28 [11] was used to trim Nextera adapters, trim low-quality sequence read-ends with base quality scores < 35, and discard reads < 100 bp. The Geneious aligner was used to align cleaned sequence reads to the 5,967 bp *Plasmodium falciparum* mitochondrial reference genome (NCBI accession M76611; identical to accession CP017005, strain 3D7), using medium sensitivity and a minimum mapping quality score of 30. Variants were identified in a region 50 bp downstream and upstream from the 5’ and 3’ ends of the reference sequence, respectively, using a threshold of ≥ 5X read depth and ≥ 10% frequency. The variant calling region was designated to reduce low-confidence variant calls in regions with lower read depth.

Suspected multi-species mixed- and/or non-*P*. *falciparum* infected samples were aligned to the following *Plasmodium* mitochondrial reference genomes: *Plasmodium vivax*, NCBI accession NC_007243; *Plasmodium ovale curtisi*, accession HQ712052; *Plasmodium ovale wallikeri*, accession HQ712053; *Plasmodium malariae*, accession LT594637; and *Plasmodium knowlesi*, accession NC_007232. Variants were identified as stated above.

**Fig 1:**
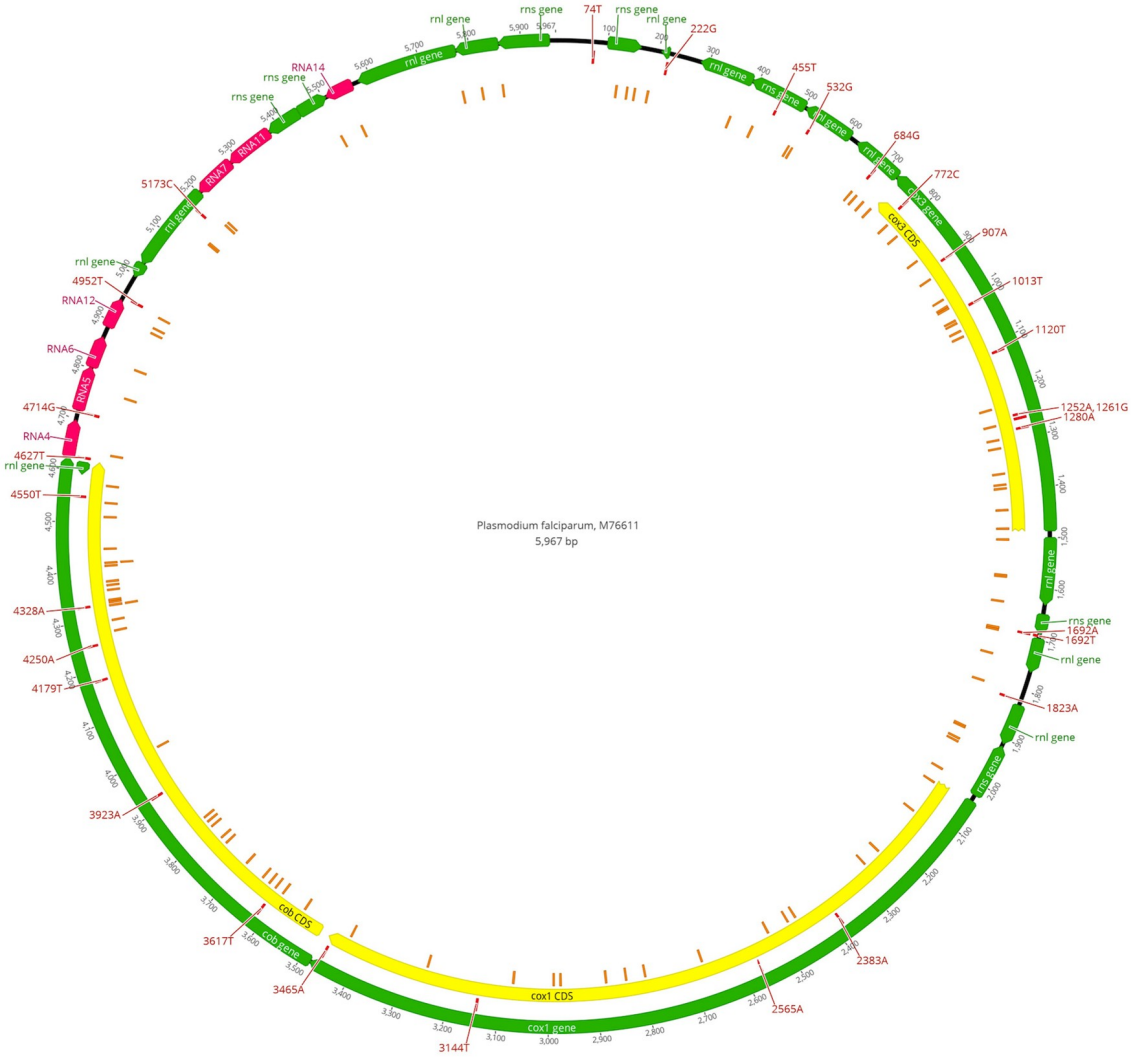
Observed single-nucleotide polymorphisms (SNPs) mapped to the *Plasmodium falciparum*, 3D7 (M76611) reference. A total of 135 variants, including 133 SNPs and 2 indels (insertions) were identified in 256 *P*. *falciparum* infected samples. Private SNPs (i.e., present in only one sample) accounted for 105/135 variants found. Orange rectangles = private SNPs. Red rectangles with labels = SNPs present in more than one sample. Additional annotations were reported from NCBI Genbank: cox3 = cytochrome c oxidase subunit 3; cox1 = cytochrome C oxidase subunit I; cob = apocytochrome b; rnl = large subunit ribosomal RNA; rns = small subunit ribosomal RNA; RNA = miscellaneous RNA.

Mitochondrial haplotypes were identified for *P*. *falciparum* infections in samples with all variants that had greater than 95% frequency. Samples with genotype allele frequencies less than 95% were considered mixed infections and were excluded during the construction of haplotypes. Sequence data (raw fastq files) can be accessed through NCBI BioProject accession PRJNA428490 [12]. Fig 1 depicting identified SNPs mapped to the *P*. *falciparum* reference genome was constructed using Geneious v11.0.5.

### Phylogenetic analysis

Maximum likelihood phylogenetic trees comprised of domestic, imported samples with unknown travel history, known travel history, and publicly available sequences with known country of origin were constructed to infer potential origin or phylogenies of samples with unknown geographical origin. Publicly available *P*. *falciparum* full mitochondrial genome sequences (n = 745) were downloaded from NCBI Nucleotide [13], with geographical origins from the following reported regions: Africa, Brazil, Central America, Ghana, Haiti, India, Papa New Guinea, Philippines, Solomon Islands, South America, Southeast Asia, Tanzania, Thailand, and Vanuatu [6,8,9,14–16].

Consensus sequences from alignments to the *P*. *falciparum* mitochondrial reference genome were hard-trimmed 50 bp on the 5’ and 3’ ends to remove low-quality bases. Multiple sequence alignments (MSAs) were constructed using MUSCLE v3.8.425 [17] for each sample with unknown travel history, domestic samples with known travel history (n = 167) and public sequences with known country of origin (n = 745). MSAs were manually trimmed to ensure all sequences in each alignment were the same length. Phylogenetic trees were constructed for each MSA using FastTree v2.1.5 [18] and iTOL [19].

### Haplotype network construction

A median-joining network was constructed using 408 mitochondrial genomes with known geographical origin (n = 343) and samples with unknown travel history (n = 65). The number of samples was reduced (from the total used previously for phylogenetic analysis) for better visualization by removing singleton haplotypes (i.e., haplotypes only occurring once), removing samples with multiple country origins, and reducing the number of samples per region per haplotype to a maximum of 10 samples. Countries/regions of origin of known samples were reclassified into 12 different geographical regions (i.e., Central Africa, Southern Africa, West Africa, East Africa, South America, Southeast Asia, Solomon Island, Papua New Guinea, Vanuatu, Haiti, Philippines, and India).

Multiple sequence alignments (MSAs) were constructed using MUSCLE v3.8.425 [17]. Haplotype data using the MSAs were generated using DNASP v6.12.01 [20], and the haplotype network was constructed using Popart v1.7 with the median-joining algorithm [21].

## Results

### *P*. *falciparum* mitochondrial haplotypes in samples imported to the U.S.

The MaRS targeted-amplicon deep sequencing protocol [4] was used to sequence the full mitochondrial genomes of 265 US domestically-imported malaria samples submitted to the CDC malaria surveillance network from 2014–2017. A total of 1,380,770 reads aligned to the *P*. *falciparum* mitochondrial reference genome for 256 *P*. *falciparum* infected samples (251 *P*. *falciparum* and 5 mixed infection samples), with a mean of 5394 (±4831) reads aligned. The average read depth for the full mitochondrial genome ranged from 15.76X to 877.10X with a mean of 176X (±152.15) per sample. A total of 135 variants, including 133 SNPs and 2 indels (insertions), were identified (Fig 1, S1 File). The number of variants per sample ranged from 0 to 6 with an average of 2 (±1) variants per sample. The most prevalent SNP, T772C, was present in 254/256 samples, and 105 variants were private SNPs (i.e., present in only one sample).

Mitochondrial haplotypes were constructed for *P*. *falciparum*-infected samples with genotype allele frequencies greater than 95% frequency. Seventy-six *P*. *falciparum* mitochondrial haplotypes were constructed for 209/256 samples (S2 File), and 47/256 of *P*. *falciparum* infected samples contained at least 2 mitochondrial genome haplotypes, indicating either heteroplasmy or multi-clonal infections (Table 1). Of the 76 haplotypes identified, 65 were private haplotypes (i.e., only observed in one sample), and 60% of all samples (n = 125/209) were representative of only 2 haplotypes, with variants 772C and 772C-1692A.

**Table 1:** Summary of *P*. *falciparum* mitochondrial haplotypes and multi-clonal infections imported to the U.S. from 2014–2017.

Travel History						
Region	Country	Samples	Haplotypes	Mixed-Species Infections	Non-Pf Infections	Heteroplasmy/Multi-clonal Infections
Africa	Angola	2	1	-	-	-
	Burkina Faso	3	2	-	-	1
	Cameroon	11	6	-	1	2
	Central African Republic	1	1	-	-	-
	Democratic Republic of the Congo	8	4	1	1	-
	Ethiopia	2	1	-	1	-
	Gabon	2	1	-	-	-
	Ghana	9	7	-	1	1
	Guinea	8	3	1	-	4
	Kenya	7	2	-	-	1
	Liberia	31	13	-	1	6
	Malawi	1	-	-	-	1
	Mali	2	2	-	-	-
	Mozambique	3	1	-	-	1
	Nigeria	28	9	1	-	4
	Rwanda	1	1	-	-	-
	Senegal	2	2	-	-	-
	Sierra Leone	15	8	-	-	2
	South Africa	1	1	-	-	-
	South Sudan	1	-	-	-	1
	Tanzania	4	2	-	-	-
	Togo	3	2	-	-	1
	Uganda	9	4	-	-	1
Caribbean	Haiti	2	2	-	-	-
Asia	Cambodia	2	1	-	-	-
Multiple Countries	Multiple Countries	14	5	-	-	3
Unknown	Unknown	93	27	3	3	18
**Total**		**265**	76*	**6**	**8**	**47**

*Number of distinct haplotypes for all samples

### Mixed-species and non-*P*. *falciparum* infections

Full mitochondrial genomes generated by MaRS were used for species identification and to resolve mixed-species infections for all 265 samples used in this study. Samples suspected of mixed infections or non-*P*. *falciparum* infections, based on visual analysis of alignments to the *P*. *falciparum* mitochondrial genome (evident by >20–400 SNPs), were aligned to mitochondrial reference genomes for *P*. *falciparum*, *P*. *vivax*, *P*. *malariae*, *P*. *ovale curtisi*, *P*. *ovale wallikeri*, and *P*. *knowlesi*.

Six samples were identified as mixed-species infections containing *P*. *falciparum* and *P*. *ovale curtisi*, *P*. *ovale wallikeri*, or *P*. *malariae* as a co-infector. All mixed-infections were able to be deconvolved into their major and minor species contributors based on read count ratios (Table 2). Five of the six mixed infections contained *P*. *falciparum* as the major species, with *P*. *falciparum* specific reads ranging from 57.3% to 97.4% of the total reads. One sample contained *P*. *malariae* as the major species with *P*. *falciparum* only comprising 7% of the total reads. The major *Plasmodium* species reads aligned to greater than 99.0% of the corresponding mitochondrial genome with mean read depths ranging from 34.8X (±17.4) to 159.5X (±78.7). The minor *Plasmodium* species for all samples ranged from 2.6% to 42.7% abundance and reads aligned to 32.9% to 93.7% of the reference mitochondrial genomes with mean read depths ranging from 0.8X (±1.3) to 107.4X (±88.3).

**Table 2:** Mixed-species malaria infections deconvolved using the *Plasmodium* mitochondrial genome.

Sample	Species		Aligned Reads		% Coverage		Average Read Depth		Travel History
	Major	Minor	Major	Minor	Major	Minor	Major	Minor	
16–182	*P*. *falciparum*	*P*. *ovale wallikeri*	4,865	347	99.6	87.2	158.9 ± 64.9	11.5 ± 10.9	Unknown
16–225	*P*. *falciparum*	*P*. *ovale wallikeri*	4,252	3,169	99.5	93.3	135.7 ± 100.9	107.4 ± 88.3	Guinea
5702	*P*. *falciparum*	*P*. *ovale wallikeri*	4,329	1,220	99.4	89.6	159.5 ± 78.7	46.9 ± 43.2	Nigeria
17–035	*P*. *malariae*	*P*. *falciparum*	3,856	292	100.0	97.0	125.8 ± 58.9	9.1 ± 6.6	Unknown
16–047	*P*. *falciparum*	*P*. *malariae*	1,089	30	99.6	32.9	34.8 ± 17.4	0.8 ± 1.3	DR Congo
17–037	*P*. *falciparum*	*P*. *ovale curtisi*	4,358	116	99.6	66.6	142.7 ± 64.2	3.8 ± 4.7	Unknown

Eight samples were identified as non-*P*. *falciparum* infections (Table 3). Reads aligned to greater than 99.0% of each *Plasmodium* reference mitochondrial genome and had a mean read depth of 62.2X (± 25.9) to 491.2X (± 205.4). *P*. *ovale wallikeri* infected samples, 17–012 and 5909, had an identical mitochondrial haplotype (i.e., 832T-1234C-1327T-1388T-1560T-1802A-2209A-2755T-3158C-3758A-5280T) as did *P*. *malariae* infected samples, 16–209 and 5910 (i.e., 431d-436A-437T-456d-670d-738+T-989d-3095+AT).

**Table 3:** Non-*P*. *falciparum* infections identified using the *Plasmodium* mitochondrial genome.

Sample	Species	Aligned Reads	% Coverage	Avg. Read Depth	# SNPs and Indels	Travel History
16–016	*P*. *ovale curtisi*	1,944	99.0	62.2 ± 25.9	8	Unknown
16–209	*P*. *malariae*	7,630	99.8	246.5 ± 96.8	7	Unknown
17–012	*P*. *ovale wallikeri*	7,238	100.0	235.8 ± 101.7	11	Ghana
5860	*P*. *vivax*	10,802	100.0	381.6 ± 200.6	3	Cameroon
5908	*P*. *vivax*	4,408	100.0	143.3 ± 63.5	3	Unknown
5909	*P*. *ovale wallikeri*	15,249	100.0	491.2 ± 205.4	11	DR Congo
5910	*P*. *malariae*	6,375	100.0	205.0 ± 92.2	7	Liberia
5914	*P*. *vivax*	13,355	100.0	421.1 ± 201.8	4	Ethiopia

### Inference of geographical origins of *P*. *falciparum* infections with unknown travel history

**Fig 2:**
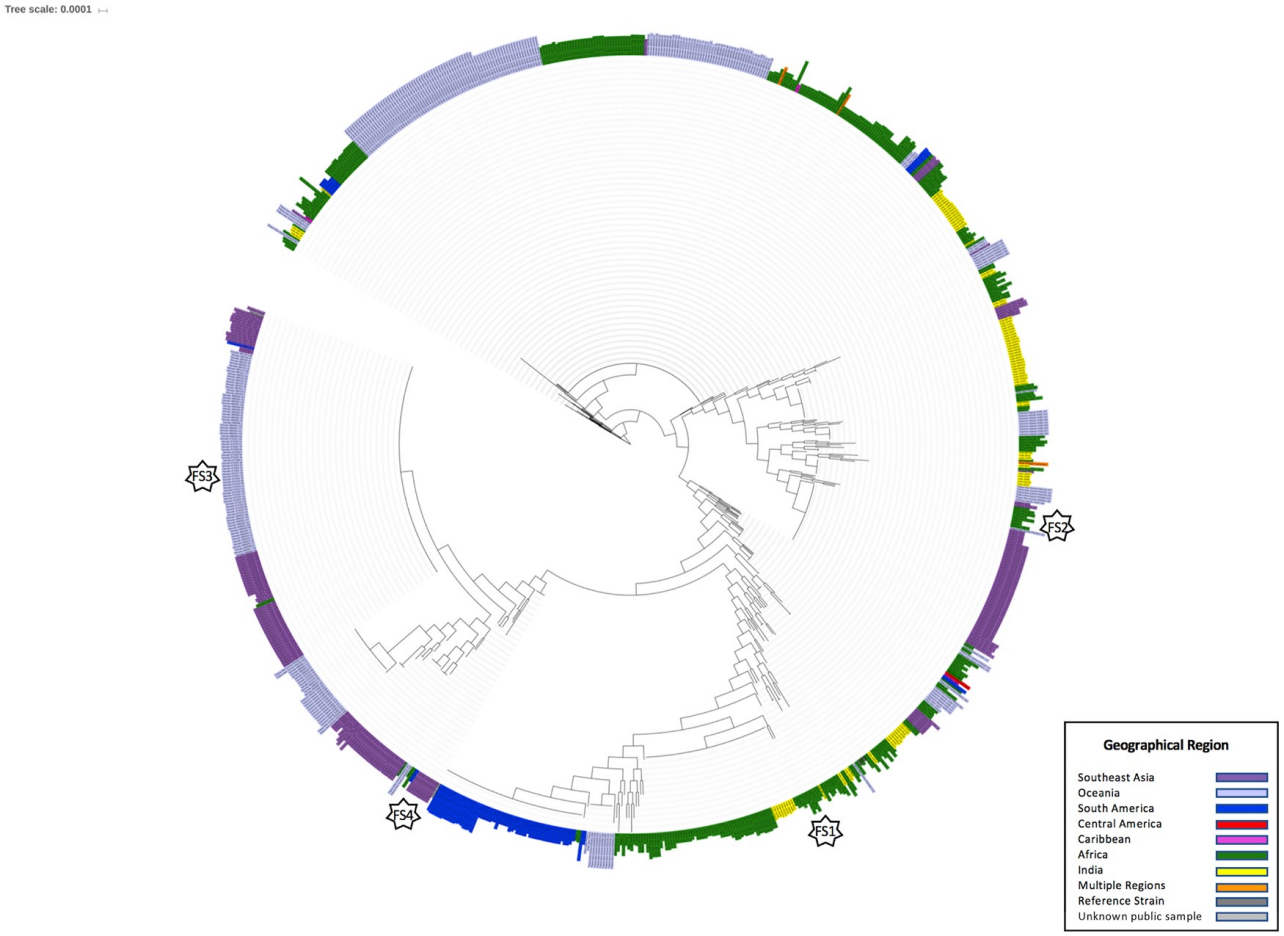
Maximum-likelihood phylogeny of 912 *Plasmodium falciparum* mitochondrial genomes with known geographical origin. A maximum-likelihood phylogenetic tree was constructed using 745 publicly available *P*. *falciparum* mitochondrial genomes and 167 *P*. *falciparum* mitochondrial genomes from US domestically imported samples with reported travel history. Purple = Southeast Asia; Light purple = Oceania; Blue = South America; Red = Central America; Pink = Caribbean; Green = Africa; Yellow = India; Orange = Multiple regions; Dark Grey = Reference strain; Light Grey = Unknown public samples. Stars labeled FS1, FS2, FS3, and FS4 correspond to the respective Supplemental Figure depicting a zoomed in section of the tree.

Travel history was only reported for 172/265 samples. Full mitochondrial genomes generated by MaRS were used to infer the geographical origin of 89 *P*. *falciparum* infected samples with unknown travel history based on the phylogenetic relationship of each unknown sample to 912 *P*. *falciparum* mitochondrial genomes with known or reported geographical origin (including 745 publicly available genomes [6,8,9,14–16] and 167 imported samples to the U.S. with known reported travel history used in this study (mixed-species samples were excluded)). The reported geographical origins of the reference *P*. *falciparum* mitochondrial genomes, include Africa, Brazil, Central America, Ghana, Haiti, India, Papa New Guinea, Philippines, Solomon Islands, South America, Southeast Asia, Tanzania, Thailand, and Vanuatu (Fig 2).

Geographical origin was inferred if an unknown sample belonged to a clade with genomes common to a particular geographical area (e.g., Africa, Southeast Asia, country-specific, etc.) Geographical origin was resolved for 13/89 samples to clades unique to Africa (n = 10), Ghana (n = 1), Southeast Asia/Oceania (n = 1), and the Philippines (n = 1) (S1–S4 Figs). An additional 4 samples shared identical sequences to samples from Guinea, Cameroon, Rwanda, and Africa (no country designation); however, each case only included one or two samples each in our reference database for comparison. The consensus sequences of 54 samples with unknown travel history were representative of the 772C and 772C-1692A haplotypes, present worldwide and in Africa and India, respectively. Additionally, geographical origin was inferred for two publicly available sequences with unknown/undocumented origin, isolates 1905 and P31 [6], to South America and Southeast Asia/Oceania, respectively (Fig 2).

**Fig 3:**
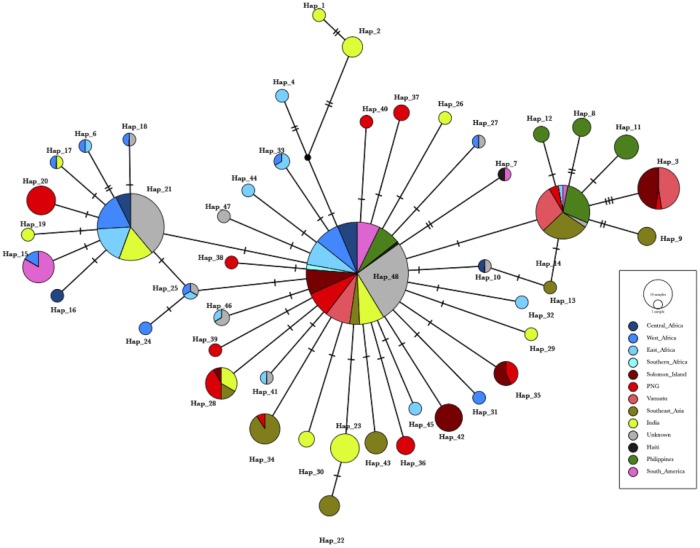
Median-joining network of 408 *Plasmodium falciparum* mitochondrial genome haplotypes from varying geographical regions. The haplotype network was estimated using 343 *P*. *falciparum* mitochondrial genomes with known geographical origin, and 65 genomes with unknown geographical origin. Each dash mark indicates the number of mutations differing between the haplotypes. PNG = Papua New Guinea. Note: Haplotype 49, comprising 9 samples from the Philippines and Unknown 5923 is not depicted as a separate haplotype due to PopArt’s masking of alignment sites with gaps. Haplotype 49 differs from Haplotype 48 with an AT insertion at site 710.

A median-joining network also was used to analyze the relationships of the more common haplotypes (i.e., occurring ≥ 2 times) based on varying geographical regions to better visualize the number of mutational steps and connections between the haplotypes observed (Fig 3). Forty-nine distinct haplotypes (204 haplotypes including singletons) were identified across 12 geographical regions from 408 samples (S3 File). Each haplotype step was separated by one to three mutations. Haplotype 48 and Haplotype 21 represent the 772C and 772C-1692A haplotypes, previously described above, respectively. Haplotype 48 represents a worldwide haplotype, present in all geographical regions mapped, and Haplotype 21 was found in Africa and India, both consistent with phylogenetic analysis. Unknown samples could be attributed to regions including Central Africa, West Africa, East Africa, the Philippines (S3 File), Southeast Asia/Oceania, and to global haplotypes, consistent with phylogenetic analysis. Twenty-eight different haplotypes were specific to a single geographical region. The haplotype network depicts a wide global distribution and connection of the haplotypes by 1 to 3 mutational steps but also shows clear clusters of haplotypes specific to Southeast Asia and Oceania (branching from Haplotype 14) and Africa and India (branching from Haplotype 21).

## Discussion

Here, we describe the utility of using the MaRS targeted-amplicon deep sequencing assay [4] to sequence indiscriminately the full *Plasmodium* species mitochondrial genome. One of the advantages of deep sequencing the *Plasmodium* mitochondrial genome is that is allows for species identification, including mixed-species infections. Targeted-amplicon deep sequencing provides a more comprehensive approach for molecular characterization of *Plasmodium* species infection by providing a high-throughput, quantitative method for species and mixed-species infection identification and improved detection of minor alleles present within a sample [4].

Sequenced full mitochondrial genomes were used to be able to classify *Plasmodium* spp. for single-species and mixed-species infections and characterize all *P*. *ovale* samples down to the subspecies level (i.e., *P*. *ovale curtisi* and *P*. *ovale wallikeri*). Minor contributing species could also be detected in mixed-infections even when more than 97% of sequencing reads were from the major infecting *Plasmodium* species. Future studies will be needed to determine the limit of detection of a targeted-amplicon deep sequencing approach and to further assess the comparison of sensitivity and specificity of molecular targets within the MaRS assay to other molecular diagnostic methods.

Additionally, a targeted-amplicon deep sequencing approach provides a high-throughput, quantitative method to measure ratios of mixed sequences within a given sample and enables extraction of sequence reads from mixed-species infection. In contrast, an analog method, such as Sanger sequencing, would require at least 10 separate PCRs and sequencing reactions (~600 bp in size) to produce two sequence reads for each sample. Alternatively, using the deep sequencing approach used in this study can produce 5394 (±4831) reads at 176X (±152.15) depth per sample for complete coverage of the mitochondrial genome for a single PCR reaction and next-generation sequencing library preparation. For all mixed-species infections tested, we were able to extract full mitochondrial genome haplotypes of the major *Plasmodium* species from each sample and use them for downstream analyses to infer geographical origin of the parasites. *P*. *falciparum* infected samples with more than one mitochondrial genome, however, present a more challenging task in deconvolving mixtures.

Multi-clonal infections may present challenges for effective treatment when drug-resistant clones may be at low levels. More than 18% of the samples in this study contained at least two or more mitochondrial haplotypes, indicating potential multi-clonal infections; however, it is important to note that multiple mitochondrial genomes could be indicative of heteroplasmy instead of multi-clonal infections due to multi-copy number mitochondrial genomes (up to ~22 copies) per cell [22–24]. Additional single-copy nuclear or apicoplast markers would be required to help distinguish between mixed-clonal infections and heteroplasmy. One of the challenges to constructing multiple haplotypes within a mixed-clonal infection is the difficulty to phase SNPs, which was not performed and out of scope for this study as only the haplotypes in each sample were used. Short-read sequence technology (used in this study) is inefficient to accurately reconstruct haplotypes within mixtures, as read lengths are typically limited to less than 300 bp. Long-read sequencing technologies, such as those employed by Pacific Biosciences (PacBio) [25], would be required to accurately phase SNPs in order to construct full haplotypes. Short-read sequencing in conjunction with long-read sequencing has been demonstrated to accurately phase mixed human mitochondrial haplotypes, however, only for mixtures at equal ratios [26]. Efforts to construct mixed haplotypes in mixed malaria infections using short-read and long-read technology would likely still be plagued by reduced confidence in SNP calls in samples when read abundance from a minor contributor in a mixed infection is low.

Another advantage to capturing the entire mitochondrial genome for molecular surveillance of malaria is to potentially infer geographical origin of the parasite. In this study we employed a phylogenetic and median-joining network approach using the full mitochondrial genome to infer geographical origin of samples imported to the U.S. with unknown travel history, a strategy previously employed using mitochondrial genomes from *P*. *vivax* [27]. We were able to infer the origin of 13 samples to regional (i.e., Africa, Southeast Asia/Oceania) or even country-specific origins (i.e., Philippines, Ghana). However, the majority of the unknown samples (n = 54/89) were representative of two of the most common *Plasmodium falciparum* mitochondrial haplotypes, 772C and 772C-1692A, which are present worldwide and in Africa and India, respectively, making geographical origin prediction for the majority of samples imported into the U.S. impossible using the mitochondrial genome alone, a finding previously reported by Preston *et al*. [7]. The global haplotypes also were described by Rodrigues *et al*. [28], whom depicted a network with similar geographical structure with a common haplotype found mainly in Southeast Asia and Oceania and another in Africa and South Asian/India, each separated by one mutational step from the global haplotype. A common haplotype found in the Americas was separated by one mutational step from the Africa and South Asian/Indian haplotype, also reported by Rodrigues *et al*. [28].

Previously, Preston *et al*. [7] developed a 23-SNP barcode, including 5 SNPs from the *Plasmodium* mitochondrial genome and 18 SNPs from the *Plasmodium* apicoplast genome (~35kb), to differentiate parasites specific to regions of South America, West Africa, East Africa, Southeast Asia, and Oceania. The 772C and 772C-1692A mitochondrial genome haplotypes from this study correspond to the 772C-853T-973T-1283A-2383G mitochondrial SNP barcode haplotype found worldwide by Preston *et al*. [7], which was present in 261/265 of the samples used in this study. Three additional samples, with known travel history to Cambodia (n = 2) and Malawi (n = 1) were concordant with mitochondrial barcode haplotypes specific to Southeast Asia/Oceania and East Africa [7], respectively. Specifically, the two samples with travel origin from Cambodia contained the 772T allele, which Preston *et al*. identified as the most informative, containing the highest Fst compared to the other SNP loci evaluated, to differentiate isolates from Southeast Asia/Oceania compared to the other three regions [7]. One sample, from Nigeria, was discordant and contained an East Africa barcode haplotype [7]. Although the mitochondrial genome does have some use for regional and country-specific geographical inference or prediction, additional markers such as those on the apicoplast as used in Preston *et al*. [7], microsatellites [29–31], and other SNP barcoding methods as described in Daniels *et al*. [32] may be needed to improve the ability to predict the geographical origin of parasites. As sequencing technologies have increased throughput, it is now feasible to employ large-scale comparative genomics analyses across thousands of sequenced genomes to identify novel markers with high heterozygosity which may prove useful for geographical tracking of *P*. *falciparum* parasites.

The ability to predict the geographical origin of parasites for molecular surveillance of drug-resistance is imperative to be able to effectively track the spread of drug-resistant parasites. Of the 265 samples used in this study, only 65% had reported travel history. While we can report on samples with unknown origins sharing the same haplotypes in samples from particular countries or regions, we are limited in our confidence due to the numbers of samples representative from these countries in the database. In order to have increased confidence in geographical origin predication we need to have larger, more deliberate samplings from countries in order to infer origin at the country level. Public databases of sequences from parasites with different geographical origins are necessary to be able to effectively track parasites. While such databases, for this purpose, are not readily available, we hope to build a database of *Plasmodium* mitochondrial genomes as part of domestic surveillance in the U.S. and to link this data with additional genetic markers that are collected with MaRS.

## Supporting information

S1 File Variant table for all samples. (CSV)

S2 File Haplotypes table for all samples. (CSV)

S3 File Haplotypes for all samples in haplotype network. (CSV)

S1 Fig Maximum-likelihood phylogeny depicting inference of Africa origin of U.S.-imported sample 16–003 with unknown travel history. A maximum-likelihood phylogenetic tree was constructed using 912 *P*. *falciparum* mitochondrial genomes with known geographical origin to infer the geographical origin of Sample 16–003 with unknown travel history. Grey triangles represent collapsed clades for visualization purposes. Blue = clade of origin.
(TIF)

S2 Fig Maximum-likelihood phylogeny depicting inference of Ghana origin of U.S.-imported sample 5884 with unknown travel history. A maximum-likelihood phylogenetic tree was constructed using 912 *P*. *falciparum* mitochondrial genomes with known geographical origin to infer the geographical origin of Sample 5884 with unknown travel history. Grey triangles represent collapsed clades for visualization purposes. Blue = clade of origin.
(TIF)

S3 Fig Maximum-likelihood phylogeny depicting inference of southeast Asia/Oceania origin of U.S.-imported Sample 16–071 with unknown travel history. A maximum-likelihood phylogenetic tree was constructed using 912 *P*. *falciparum* mitochondrial genomes with known geographical origin to infer the geographical origin of Sample 16–071 with unknown travel history. Grey triangles represent collapsed clades for visualization purposes. Blue = clade of origin.
(TIF)

S4 Fig Maximum-likelihood phylogeny depicting inference of Philippines origin of U.S.-imported sample 5923 with unknown travel history. A maximum-likelihood phylogenetic tree was constructed using 912 *P*. *falciparum* mitochondrial genomes with known geographical origin to infer the geographical origin of Sample 5923 with unknown travel history. Grey triangles represent collapsed clades for visualization purposes. Blue = clade of origin.
(TIF)
