# Supplementary figures and images for: Using the Plasmodium mitochondrial genome for classifying mixed-species infections and inferring the geographical origin of P. falciparum parasites imported to the U.S
Source: PLoS One. 2019 Apr 30;14(4):e0215754. doi: 10.1371/journal.pone.0215754 (PMC6490880; doi:10.1371/journal.pone.0215754)

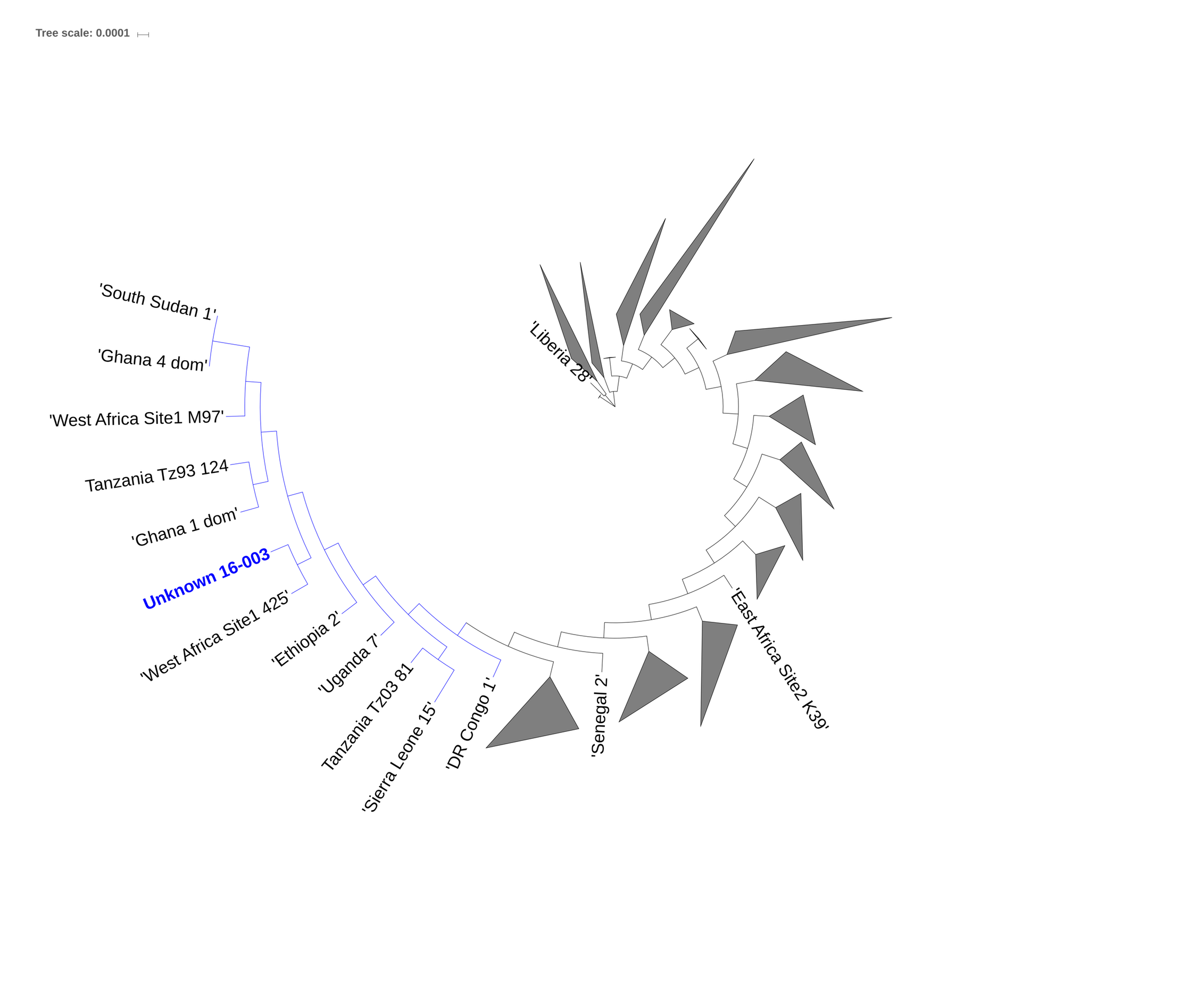

Supplement: S1 Fig — A maximum-likelihood phylogenetic tree was constructed using 912 P. falciparum mitochondrial genomes with known geographical origin to infer the geographical origin of Sample 16–003 with unknown travel history. Grey triangles represent collapsed clades for visualization purposes. Blue = clade of origin. (TIF) [file pone.0215754.s004.tif]

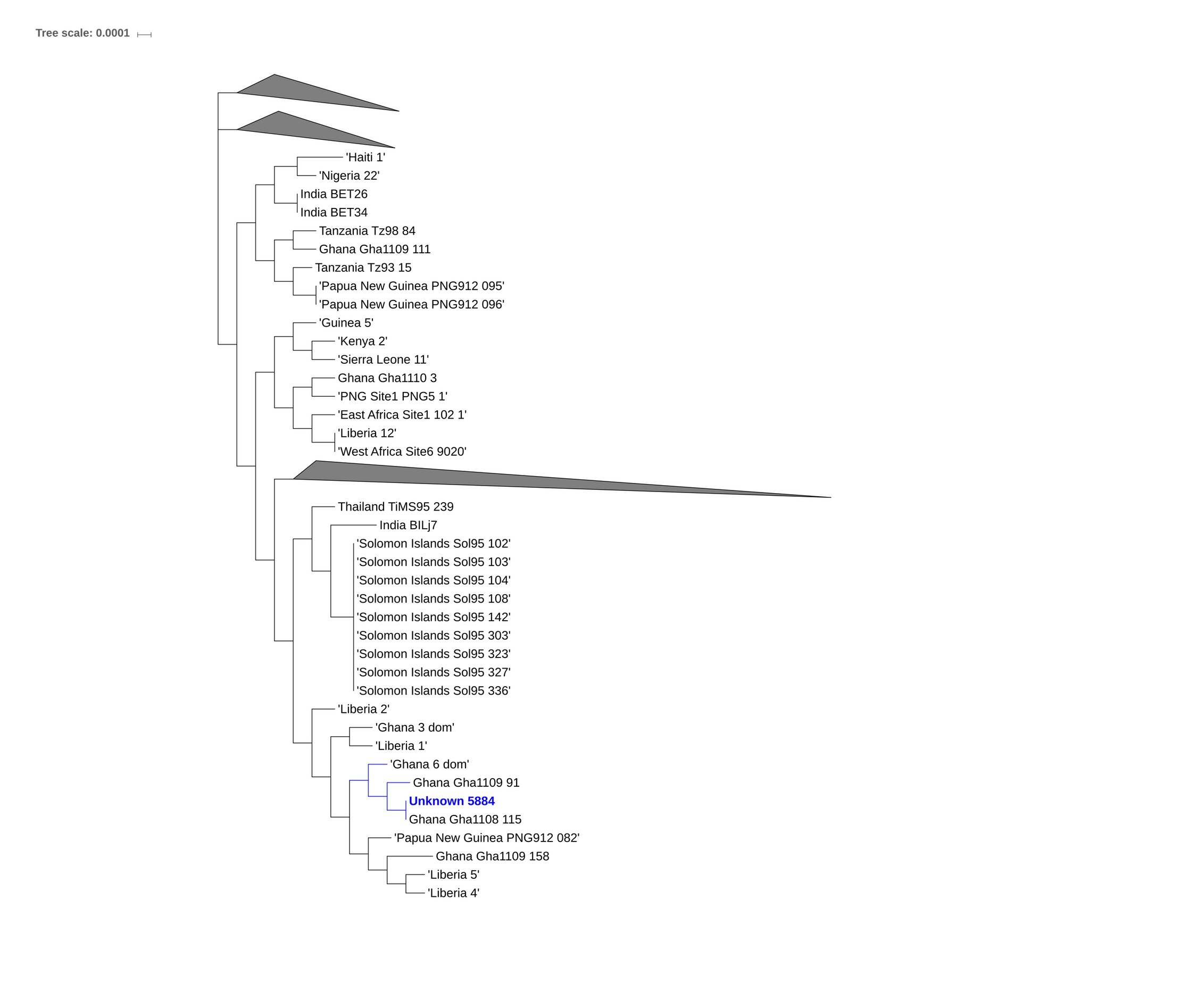

Supplement: S2 Fig — A maximum-likelihood phylogenetic tree was constructed using 912 P. falciparum mitochondrial genomes with known geographical origin to infer the geographical origin of Sample 5884 with unknown travel history. Grey triangles represent collapsed clades for visualization purposes. Blue = clade of origin. (TIF) [file pone.0215754.s005.tif]

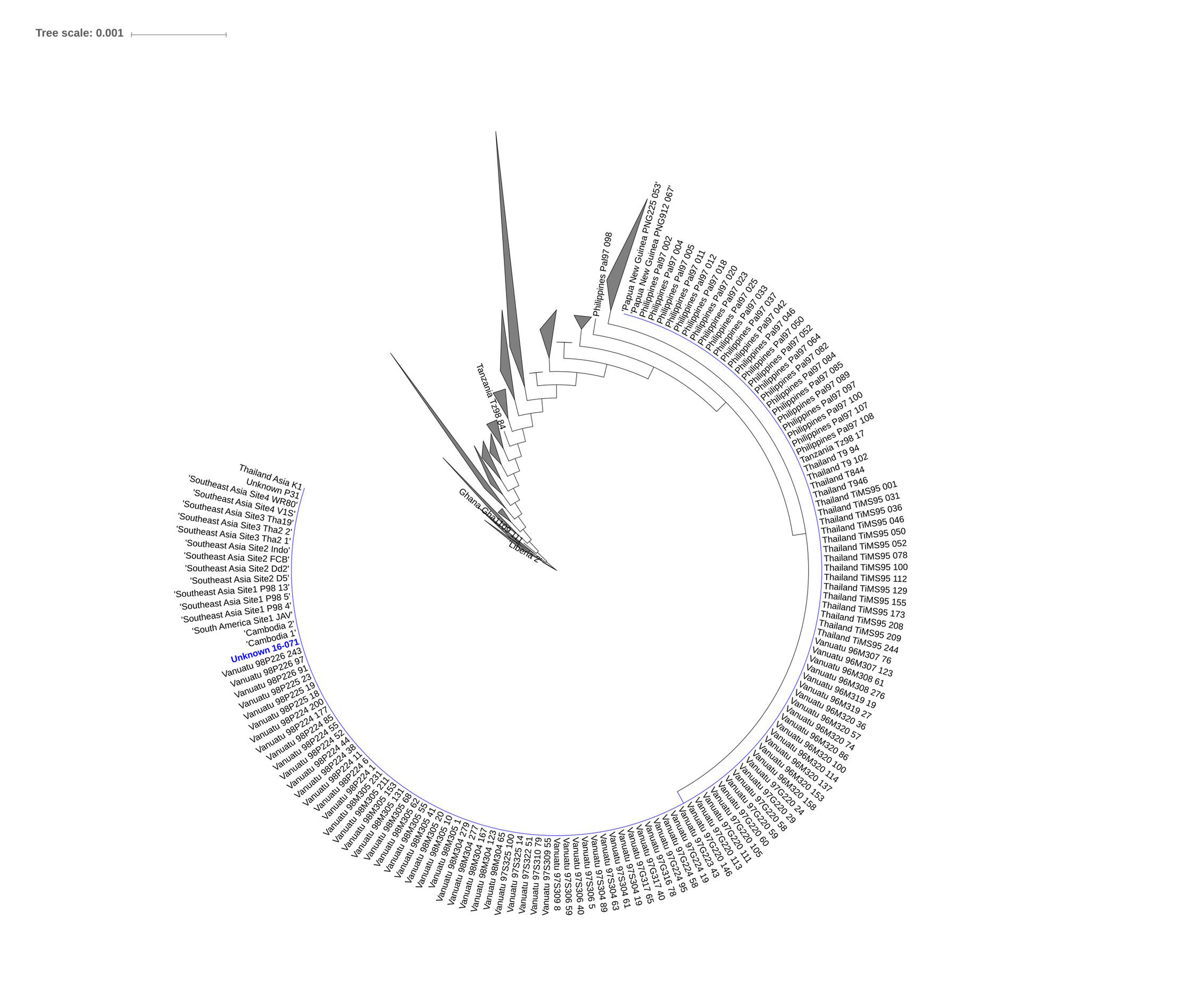

Supplement: S3 Fig — A maximum-likelihood phylogenetic tree was constructed using 912 P. falciparum mitochondrial genomes with known geographical origin to infer the geographical origin of Sample 16–071 with unknown travel history. Grey triangles represent collapsed clades for visualization purposes. Blue = clade of origin. (TIF) [file pone.0215754.s006.tif]

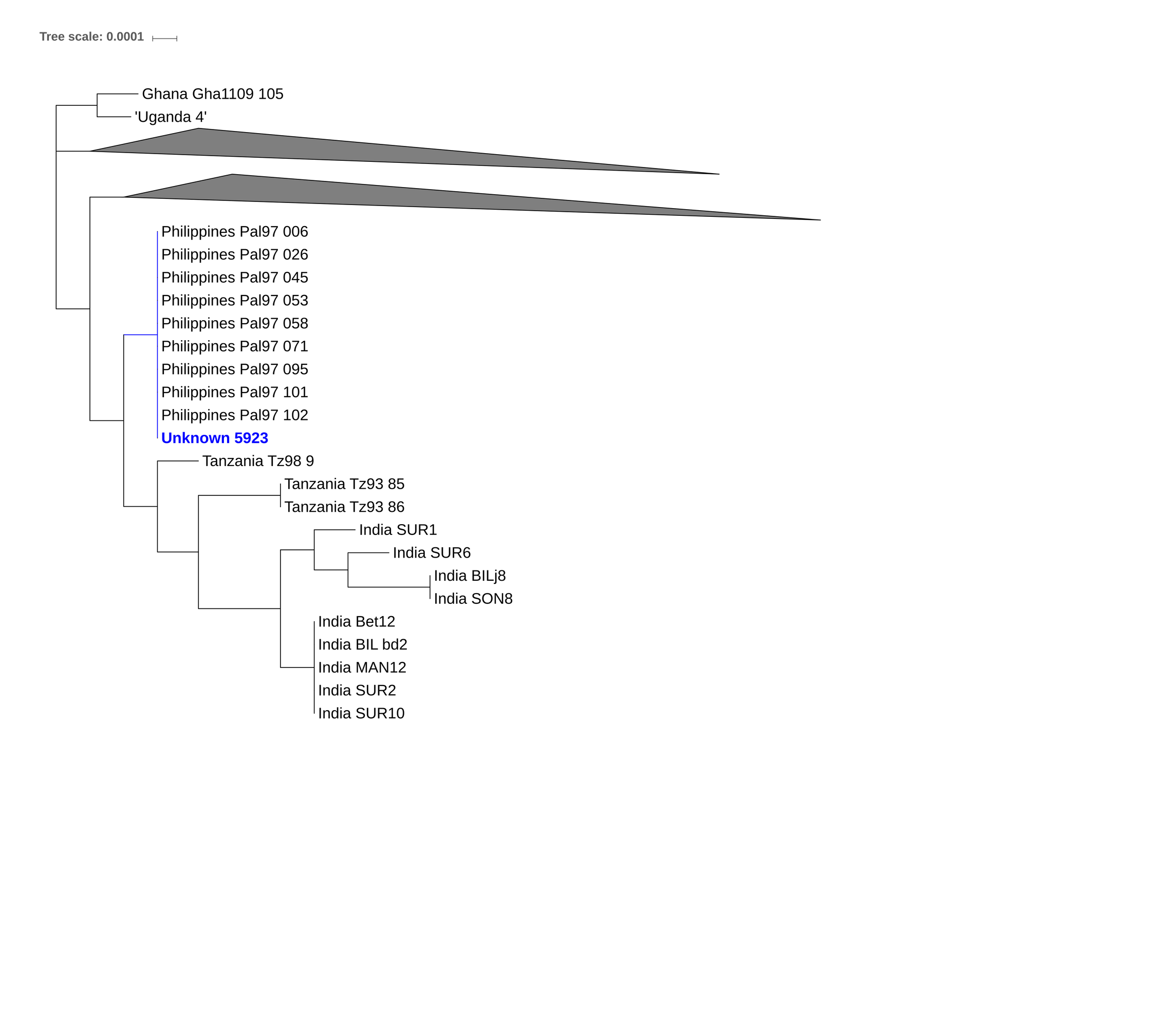

Supplement: S4 Fig — A maximum-likelihood phylogenetic tree was constructed using 912 P. falciparum mitochondrial genomes with known geographical origin to infer the geographical origin of Sample 5923 with unknown travel history. Grey triangles represent collapsed clades for visualization purposes. Blue = clade of origin. (TIF) [file pone.0215754.s007.tif]
